# Chronic High-Fat Diet Impairs Collecting Lymphatic Vessel Function in Mice

**DOI:** 10.1371/journal.pone.0094713

**Published:** 2014-04-08

**Authors:** Katrin S. Blum, Sinem Karaman, Steven T. Proulx, Alexandra M. Ochsenbein, Paola Luciani, Jean-Christophe Leroux, Christian Wolfrum, Michael Detmar

**Affiliations:** 1 Institute of Pharmaceutical Sciences, Swiss Federal Institute of Technology, ETH Zurich, Zurich, Switzerland; 2 Institute of Food, Nutrition and Health, Swiss Federal Institute of Technology, ETH Zurich, Schwerzenbach, Switzerland; University of Queensland, Australia

## Abstract

Lymphatic vessels play an essential role in intestinal lipid uptake, and impairment of lymphatic vessel function leads to enhanced adipose tissue accumulation in patients with lymphedema and in genetic mouse models of lymphatic dysfunction. However, the effects of obesity on lymphatic function have been poorly studied. We investigated if and how adipose tissue accumulation influences lymphatic function. Using a lymphatic specific tracer, we performed *in vivo* near-infrared (NIR) imaging to assess the function of collecting lymphatic vessels in mice fed normal chow or high-fat diet (HFD). Histological and whole mount analyses were performed to investigate the morphological changes in initial and the collecting lymphatic vessels. HFD was associated with impaired collecting lymphatic vessel function, as evidenced by reduced frequency of contractions and diminished response to mechanostimulation. Moreover, we found a significant negative correlation between collecting lymphatic vessel function and body weight. Whole mount analyses showed an enlargement of contractile collecting lymphatic vessels of the hind limb. In K14-VEGF-C mice, HFD resulted in a reduced spreading of the tracer within dermal lymphatic vessels. These findings indicate that adipose tissue expansion due to HFD leads to a functional impairment of the lymphatic vasculature, predominantly in collecting lymphatic vessels.

## Introduction

The lymphatic system is essential in mediating tissue fluid homeostasis, immune surveillance and uptake of dietary fat in the intestine [1]. A number of observations indicate close interactions between lymphatic vessels and adipose tissue [2]. Accumulation of adipose tissue is regularly observed in the affected tissue of lymphedema patients [3], there is a close association of lymph nodes (LNs) and collecting lymphatic vessels with adipose tissue throughout the body [4], and addition of lymph fluid to preadipocytes *in vitro* promoted their differentiation into mature adipocytes [5]. These observations are supported by findings in genetic [5]–[7] and surgical [8] mouse models of lymphedema. Mice haploinsufficient for Prox1, a master regulator of lymphatic differentiation, develop chylous ascites and adult onset obesity due to defective lymphatic vessels [5]. Moreover, in two mouse models of primary lymphedema –*Chy* [7] and K14-VEGFR-3-Ig mice [6]– an inverse correlation between dermal lipid accumulation and hydraulic conductivity was found [9]. Conversely, it remains unclear if obesity affects lymphatic function. In mice, it was recently found that obesity diminishes lymphatic fluid transport to draining lymph nodes [10]. However, in humans the data are more controversial –while one study found impairment of lymphatic function in morbidly obese patients [11], another recent report found similar lymph flow between normal weight and obese individuals [12].

We aimed to investigate if and how diet-induced obesity affects the morphology and function of the lymphatic vasculature. To this end, mice were fed a high-fat diet (HFD) and then we performed morphometric assessments of lymphatic vessels and employed high-resolution near infrared (NIR) imaging to quantitatively investigate the function of collecting lymphatic vessels in the hind-leg of mice *in vivo*. In three different mouse strains, we found that HFD was associated with an impairment of collecting lymphatic vessel function. These findings imply that lymphatic dysfunction can be induced by adipose tissue expansion.

## Materials and Methods

### 3.1 Animal models-Ethics statement

Male C57BL/6J:ICR mice (n = 20), FVB mice (n = 16) and K14-VEGF-C mice [13] (n = 8) were kept under conventional SPF conditions. For each strain, starting at 4 weeks of age, half of the mice were provided *ad libitum* access to standard chow (chow; 11% kcal from fat, 31% kcal from protein, and 58% kcal from carbohydrate; Provimi-Kliba, Kaiseraugst, Switzerland), and the other half to high-fat diet (HFD; 60% kcal from fat, 20% kcal from protein, and 20% kcal from carbohydrate; Research Diets Inc., New Brunswick, NJ, USA) for 17 weeks or 31 weeks. Mice were anesthetized by i.p. injection of 0.2 mg/kg medetomidine and 80 mg/kg ketamine for imaging. Mice were sacrificed with an overdose of anesthesia (1000 mg/kg ketamine/3.5 mg/kg medetomidine) followed by cervical dislocation. All experiments were performed in accordance with animal protocols approved by Kantonales Veterinäramt Zürich.

### 3.2 Analysis of water content

Water content was analyzed as previously described [9]. Briefly, back skin samples were collected with 6 mm biopsy punches (Acuderm, Fort Lauderdale, FL, USA). The samples were weighed after the collection and freeze-dried for 24 h. Thereafter, the samples were weighed again, and the weight loss was calculated. Since this procedure removed the water from the tissues, the weight loss represented the water content of the tissue.

### 3.3 Histology and immunofluorescence analyses

Cryosections of the tails (7 μm) were fixed for 2 min in acetone (–20°C) and for 5 min in 80% methanol (4°C), washed in PBS and incubated overnight (4°C) with a hamster anti-podoplanin antibody (clone 8.1.1, Developmental Studies Hybridoma Bank, University of Iowa) and rat anti-Meca32 antibody (1∶200, BD Pharmingen, Allschwil), or rabbit anti-LYVE-1 (1∶600, AngioBio) and rat anti-CD31 (1∶200, BD Pharmingen). The samples were then incubated for 30 min with Alexa488- and Alexa594-conjugated secondary antibodies (1∶200) and Hoechst 33342 (1∶1000; all from Invitrogen, Basel, Switzerland). Regular hematoxylin staining was used to stain paraffin sections (8 μm) of the tail.

### 3.4 Morphometric and morphologic analyses

Immunofluorescence stains of tail skin sections for podoplanin-positive lymphatic vessels and for Meca32-positive blood vessels were examined on an Axioskop 2 mot plus microscope (Carl Zeiss, Feldbach, Switzerland), and the images of 3 to 5 individual fields of view per section were acquired with an AxioCam MRc camera with a Plan-APOCHROMAT 10×/0.45 NA objective and AxioVision software 4.7.1 (Carl Zeiss, Feldbach, Switzerland). The thickness of the epidermis, the dermis and the adipose tissue was determined on hematoxylin-stained paraffin sections of tail skin. The morphometric analyses of the vessels and of tissue thickness were performed with ImageJ software [14].

### 3.5 Whole mount immunostaining

We harvested whole ears and the collecting lymphatic vessels from the lower limb for whole mount analysis after 31 weeks of either chow or HFD. Ears were split in half and the inner part of the ear (without cartilage) was used. Evans blue (5 μL, 0.1%) was injected intradermally to visualize the collecting lymphatic vessels of the hind limb to aid in the dissection of the lower limb collecting lymphatic vessels and the associated saphenous vein. The tissue samples were immobilized using insect pins onto self-made silicon-coated tissue culture plates. The tissues were then fixed with 4% PFA in PBS for 2 h and blocked with immunomix (5% normal donkey serum, 1% BSA, 0.1% Triton-X 100 and 0.05% NaN_3_) for 1 h. Rat-anti mouse CD31 (BD Pharmingen, 1∶250 dilution) and mouse-anti-mouse α-smooth muscle actin (Sigma, 1∶1000 dilution, conjugated to Cy3) were diluted in immunomix and the tissues were incubated in the primary antibody mix overnight at 4°C. After washing with PBS for 2 h, the samples were incubated with an Alexa488 conjugated donkey-anti-rat IgG (Molecular probes, 1∶200 dilution) for 2 h at room temperature. Thereafter, the samples were washed with PBS for at least 2 h and mounted on glass slides using Mowiol (Calbiochem). Confocal imaging was performed on a Zeiss LSM 710-FCS confocal microscope equipped with a 20× 0.8 NA Plan-Apochromat objective (Carl Zeiss). Z-stack images were acquired using Zeiss ZEN 2009 software and processed using ImageJ [14].

### 3.6 *In vivo* imaging of lymphatic vessel function

A Zeiss StereoLumar.V12 stereomicroscope adapted for NIR visualization was used for lymphatic imaging [15]. Videos were recorded during intradermal injection of the lymph-specific tracers P20D680 or P40D680 into the foot [15]. Additional videos to visualize the contractility of collecting lymphatic vessels draining the lower limb were acquired before and after mechanostimulation of the injection site. Mechanostimulation was performed using a cotton swab pressed gently on the injection site once per second for ten seconds to stimulate initial lymphatic uptake of the tracer and demonstrate the capacity of the lymphatic system to react to a sudden fluid load [15]. A region of interest (ROI) analysis of fluorescence intensity was performed to assess the frequency of collecting lymphatic vessel contractions. The response to mechanostimulation was assessed by determining the fold-change in mean fluorescence intensity during 30 s periods immediately before mechanostimulation and starting from 30 s after mechanostimulation.

### 3.7 Perfused skin area analysis

Two to three NIR images of the hind limb were combined together to obtain an overview of the whole leg – including the ankle and the distal third of the upper leg – using Photoshop software v 5.0 (Adobe Systems). A rectangular ROI of 6 mm in width and 15 mm in height was placed on the NIR picture, taking the midpoint of the arc of the dorsal collecting lymphatic vessel as a reference point, and the image was cropped. The total area of the skin and the dye-perfused area within the cropped image were then measured by manual selection using the polygonal lasso tool of Photoshop software. The perfused area was then calculated as a percentage of the total area of skin.

### 3.8 Statistical analyses

All data are shown as mean ± SD. Means of groups were compared using Student's *t*-test; Welch's correction was used in case of unequal variances, and Mann-Whitney *U* test was used for the comparisons of non-normally distributed data. P<0.05 was accepted as statistically significant.

## Results

### 4.1 Weight gain and adipose tissue enlargement after HFD

**Figure 1:**
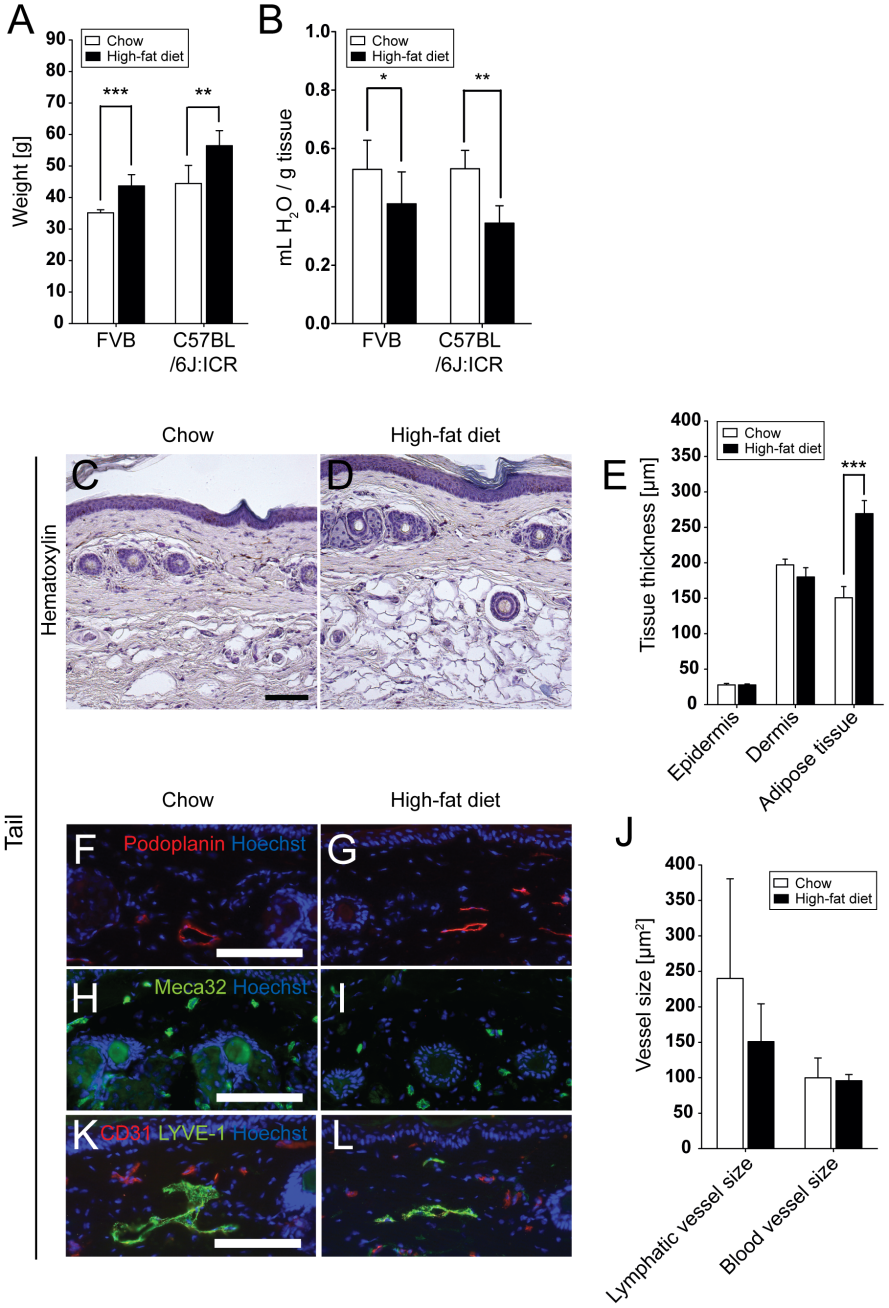
Reduced water content and increased adipose tissue after HFD. Significantly increased body weight (A) and reduced skin water content in back skin (B) after 17 weeks of HFD. Hematoxylin stainings (C,D) and quantitative image analyses (E) revealed a significantly increased thickness of the adipose tissue, but not of the epidermis or dermis, in the tail skin of C57BL/6J:ICR HFD mice. Immunofluorescence stains for podoplanin (F,G; red; lymphatic vessels) and Meca32 (H,I; green; blood vessels) and quantitative image analyses (J) revealed a reduction of the average dermal lymphatic vessel size, but not blood vessel size, after HFD. Scale bars  =  100 μm. Data represent mean ± SD. *P≤0.05, **P≤0.01, ***P≤0.001.

HFD was initiated in male FVB and C57BL/6J:ICR mice at 4 weeks of age. After 17 weeks on HFD, the body weight was significantly increased in both strains, as compared to the chow fed group (FVB: HFD, 43.7±3.6 g vs. chow, 35.2±1.0 g (n = 8); P = 0.0002; C57BL/6J:ICR: HFD, 56.5±4.8 g vs. chow, 44.5±5.7 g (n = 5); P = 0.0071; Figure 1A). The water content of back skin was reduced after 17 weeks on HFD (FVB: HFD, 0.41±0.11 mL/g vs. chow, 0.53±0.10 mL/g; P = 0.0405; C57BL/6J:ICR: HFD, 0.34±0.06 mL/g vs. chow, 0.53±0.06 mL/g; P = 0.0013; Figure 1B). A significant increase of adipose tissue with hypertrophic adipocytes (Figure 1C and D) was detected in the tails (subcutaneous layer thickness in HFD, 269.5±18.4 μm vs. chow, 150.8±15.9 μm; P<0.0001, Figure 1E).

### 4.2 Comparable initial lymphatic vessel density in skin

Immunofluorescence stains for blood vessels and lymphatic vessels were done using antibodies against podoplanin and Meca32. Morphometric analyses of blood vessels and lymphatic vessels in the tail dermis revealed that the HFD fed mice had a tendency towards reduced average dermal lymphatic vessel size (151.0±53.3 μm^2^) compared to chow fed mice (240.2±140.4 μm^2^; NS), whereas blood vessels remained unaffected (HFD, 95.8±8.8 μm^2^ vs. chow, 99.9±28.0; NS; Figure 1F-J). This tendency was also observed when the tails were stained for LYVE-1 and CD31 (Figure 1K and L).

### 4.3 Impaired collecting lymphatic vessel function in HFD fed mice

**Figure 2:**
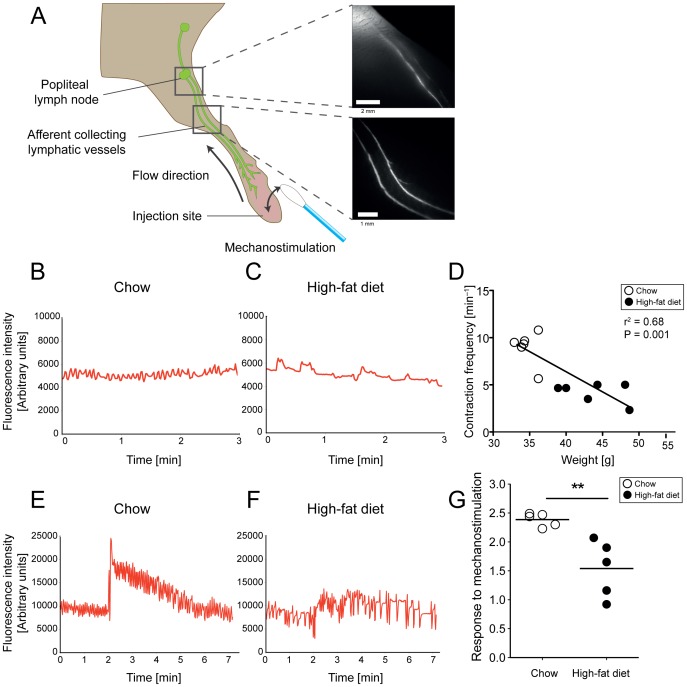
Impaired collecting lymphatic vessel function after 17 weeks of HFD in FVB mice. NIR-imaging (A) was performed after intradermal injection of 5 μL of 25 μM P20D680, a tracer specific for uptake into lymphatic vessels. Collecting lymphatic vessel contractility analysis was performed on the afferent vessels near the popliteal LN, while the response to mechanostimulation of the injection site by a cotton swab was assessed on the collecting lymphatic vessels of the lower limb. Normal (B) and irregular (C) contraction patterns in chow and HFD mice, respectively. Linear regression analysis (D) shows a significant negative correlation between weight and contraction frequencies. Normal (E) and impaired response (F) to mechanostimulation. Mice on HFD showed reduced response to mechanostimulation (G) as compared to mice on chow diet. **P≤0.01.

We next assessed the collecting vessel function in the limb of the mice using NIR imaging (Figure 2A). We first visualized and quantified the contraction frequency of the afferent collecting lymphatic vessels near the entrance to popliteal LN. We also determined the response to an acute fluid load – by mechanostimulation – of the afferent collectors in the lower limb during mechanostimulation to the injection site.

We found that contractility of collecting lymphatic vessels in the hind legs of chow fed mice appeared normal, with contraction frequencies of approximately 9 per minute (Figure 2B, Figure S1A and Video S1). In contrast, the contractions of collecting lymphatic vessels in HFD mice were without a definite pattern (Figure 2C, Figure S1A and Video S2,) and contraction frequencies were significantly reduced (chow: 9.0±1.7 contractions/min vs. HFD: 4.2±1.1 contractions/min, P = 0.0002). There was a significant inverse correlation between the weight of the mice and the contraction frequencies in both FVB (r^2^ = 0.68, P = 0.001; Figure 2D) and C57BL/6J:ICR (r^2^ = 0.52, P = 0.028; Figure S1A) strains.

We also found a reduced response to mechanostimulation in mice on HFD. Normal mice displayed a typical pattern of an immediate signal increase in the downstream collecting lymphatic vessels during mechanostimulation of the injection site, followed by a slow decrease in signal over time (Figure 2E). In contrast, most mice on HFD had a reduced signal increase during mechanostimulation and no apparent decrease in signal over time (Figure 2F). We quantified this response using the fold-change in mean fluorescence intensities of the collecting lymphatic vessels from before to after mechanostimulation. Using this assessment, we found a significant reduction in the increase of signal after mechanostimulation in collecting lymphatic vessels under HFD in both FVB (chow: 2.4±1.3 fold vs. HFD: 1.5±0.5 fold, P = 0.0079; Figure 2G) and C57BL/6J:ICR mice (chow: 2.9±0.1 fold vs. HFD: 1.2±0.2 fold, P = 0.0159; Figure S1B).

### 4.4 Enlargement of collecting lymphatic vessels in the lower limb

**Figure 3:**
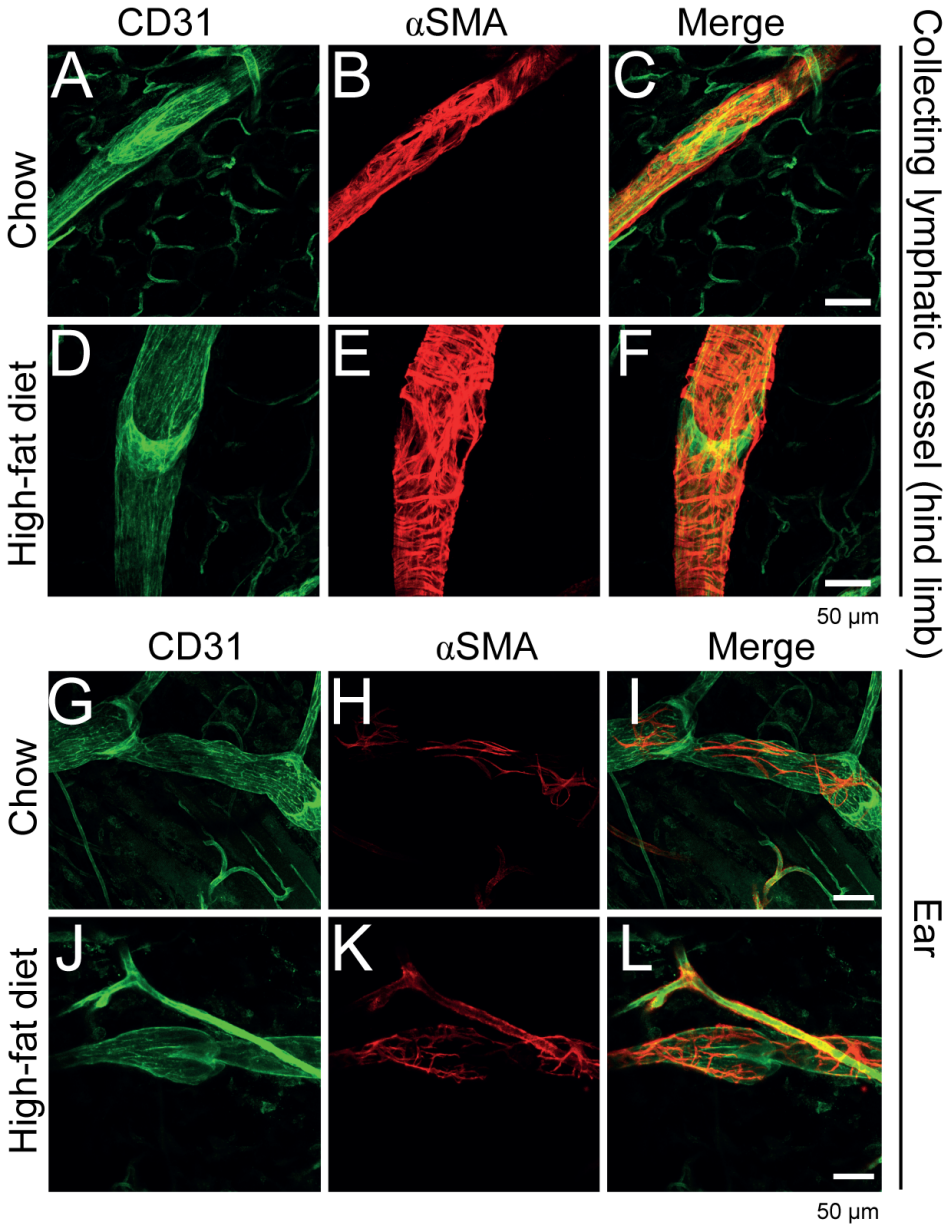
Whole mount analysis revealed enlarged collecting lymphatic vessels of the hind limb. CD31 (A, green) and αSMA (B, red) stainings of collecting lymphatic vessels from chow diet fed mice were compared to CD31 (D) and αSMA (E) stainings of collecting lymphatic vessels from HFD fed mice. The collecting lymphatic vessels were observed to be enlarged in the HFD fed mice. CD31 (G, green) and αSMA (H, red) stainings of non-contractile ear collecting lymphatic vessels from chow diet fed mice were compared to CD31 (J, green) and αSMA (K, red) stainings from HFD fed mice. No major differences were observed. Merged channels are shown in (C, F, I and L). Scale bars  =  50 μm.

We next aimed to determine if the functional differences in contractility of collecting lymphatic vessels in mice on HFD versus normal diet were associated with any morphological changes in the collecting lymphatic vessels of the lower limb. For this experiment, we fed C57BL/6J:ICR mice either HFD or chow for 31 weeks. We imaged the collecting lymphatic vessels of the mice for the above parameters and found similar reductions in contraction frequency and lack of response to mechanostimulation (data not shown). After imaging, we sacrificed the mice and harvested the collecting lymphatic vessels of the lower limb. We performed whole mount immunostaining for CD31 (to visualize the lateral saphenous vein and the accompanying collecting lymphatic vessels and the lymphatic valves) and αSMA (to visualize smooth muscle cell coverage) in order to determine whether the collecting lymphatic vessel, the lymphatic valve morphology, or the smooth muscle cell coverage were affected [16]. We found no obvious differences in the smooth muscle cell coverage as well as the general valve structure between the chow and HFD collecting lymphatic vessels. Interestingly, however, the collecting lymphatic vessels of the HFD mice appeared enlarged (Figure 3A-F). We also evaluated non-contractile collecting lymphatic vessels of the ear for the same parameters and observed no apparent differences in vessel diameter, smooth muscle cell coverage or valve morphology (Figure 3G-L).

### 4.5 Decreased spread of tracer in dermal lymphatic vessels in K14-VEGF-C mice after HFD

**Figure 4:**
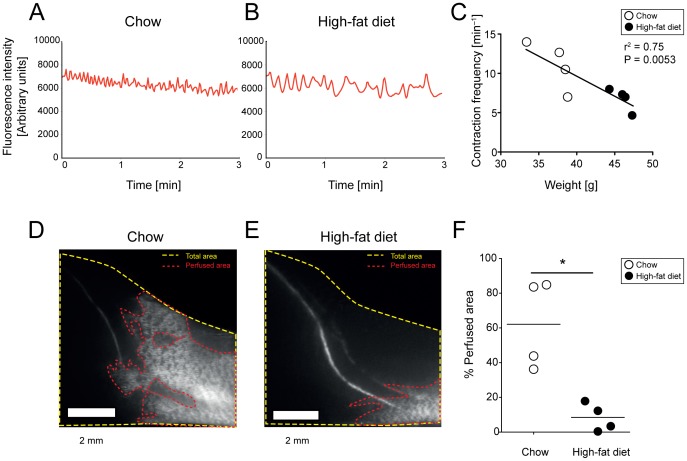
Impaired collecting lymphatic vessel function and reduced spread of tracer in dermal lymphatic vessels after 17 weeks of HFD in K14-VEGF-C mice. NIR-imaging was performed after intradermal injection of 5 μL of 25 μM P20D680. Normal (A) and irregular (B) contraction patterns in chow and HFD mice, respectively. Linear regression analysis (C) shows a significant negative correlation between weight and contraction frequencies. High (D) and low (E) spreading of tracer within dermal lymphatic vessels in chow and HFD fed mice, respectively, after 15 minutes of imaging. Scale bars  =  2 mm. Quantification (F) shows lower percentage of tracer-perfused lymphatic vessels of the skin in the lower limb of mice on HFD as compared to chow diet. *P≤0.05.

K14-VEGF-C mice have an expanded dermal lymphatic vessel network and display an increased spreading of tracers through these vessels from an intradermal injection site (Figure S2) [13], [17], [18]. These mice also have enlarged collecting lymphatic vessels in the skin compared to wildtype mice (data not shown). We hypothesized that HFD-induced expansion of subcutaneous adipose tissue may alter the spread of the lymphatic tracer in the initial dermal lymphatic vessels of K14-VEGF-C mice. Similar to the results in wild-type mice, we found irregular collecting lymphatic vessel contraction frequencies in K14-VEGF-C mice on HFD (Figure 4A-C), with a significant inverse correlation between the frequency of lymphatic contractions and body weight (r^2^ = 0.75, P = 0.0053). K14-VEGF-C mice under chow diet exhibited a pronounced spread of the lymphatic tracer throughout the expanded dermal lymphatic vessels (Figure 4D and Video S3). In contrast, the spread of the tracer was reduced under HFD conditions (Figure 4E and Video S4), resulting in a significant difference in skin area that is perfused with tracer in dermal lymphatic vessels between chow and HFD-fed mice (chow: 62.1±25.7% vs. HFD: 8.5±8.0%, P = 0.0286; Figure 4F).

## Discussion

The present study reveals morphological and functional changes of collecting lymphatic vessels in extremities of obese mice. With NIR lymphatic imaging of collecting lymphatic vessels, we found a decreased frequency of contractions and reduced response to mechanostimulation in three different mouse strains fed HFD. Morphologically, contractile collecting lymphatic vessels of the hind limb appeared larger in mice fed HFD compared to chow fed mice. In conjunction with our findings of reduced spread of tracers in dermal lymphatic vessels in HFD-fed K14-VEGF-C mice, these findings suggest that an increased subcutaneous adipose tissue expansion in response to HFD may be detrimental to lymphatic transport. These findings are in agreement with the recent report of reduced lymphatic transport in mice fed high-fat diet using PET-CT imaging techniques [10]. Our results indicate that this reduced transport capacity may be in part due to effects on the collecting lymphatic vessels, in addition to structural changes within draining lymph nodes suggested in a previous study [10].

Dilated collecting lymphatic vessels with a reduction in the frequency of contractions have been found in experimental models of inflammation and cancer [15], [19]. Similar to our current findings, a reduction in the contraction frequencies of the mesenteric collecting lymphatic vessels was found in a rat model of metabolic syndrome [20]. Since it is well established that obesity is associated with low-level inflammation, there may be direct effects of inflammatory cytokines on lymphatic vessels during obesity [10], [21]. Functionally, collecting lymphatic vessels under HFD conditions exhibited a decreased frequency of contractions and a diminished response to mechanostimulation. Given that lymphangions, the contractile units of collecting lymphatic vessels, function similar to heart ventricles [22], we assume that decreases in regularity and frequency of contractions could result in reduced lymphatic flow, as has been found in humans with lipedema [23] and in obese mice [10]. Supporting this conclusion, a decreased response to mechanostimulation of the injection site indicates a diminished tracer perfusion into the collecting lymphatic vessels, implying a higher resistance to flow inside the collecting lymphatic vessel similar to the changes suggested for veins in obese patients [24].

A typical consequence of impaired or obstructed lymphatic flow is dermal backflow, which describes the retrograde flow of injected dye from the collecting lymphatic vessels into the initial dermal lymphatic vessels due to high intravascular pressure and valve deficiency [15], [25]. Despite signs of collecting lymphatic vessel dysfunction, dermal backflow or collateral rerouting were not observed after 17 weeks of HFD. Our finding of reduced water content in the skin tissue is also not supportive of the development of lymphedema at this time point, unlike the findings in hypercholesterolemic mice where paw swelling was present [26]. When we performed imaging in K14-VEGF-C transgenic mice, which show dramatic dermal lymphatic vessel expansion and rapid spreading of lymphatic tracers after intradermal injection, we found decreased dermal lymphatic vessel spreading under HFD conditions. These findings suggest a model that expanded subcutaneous fat and reduced skin water content may have detrimental consequences for lymphatic transport through elevation of the tissue pressure in the dermis and/or diminished hydraulic conductivity of the flow within dermal lymphatic vessel [9]. Further studies are necessary to evaluate this hypothesis. It will also be of interest to investigate whether the impairment of lymphatic vascular function might be reversible after a reduction of the calorie intake.

## Supporting Information

Figure S1 **Impaired collecting lymphatic vessels function after 17 weeks of HFD in C57BL/6J:ICR mice.** NIR imaging was performed after intradermal injection of 5 μL of 25 μM P40D680, a tracer specific for uptake into lymphatic vessels. (A) Normal and irregular contraction patterns in chow (n = 5) and HFD (n = 5) mice, respectively. Linear regression analysis shows a significant negative correlation between weight and contraction frequencies. (B) Normal and impaired response to mechanostimulation. Videos were initiated 15 s after mechanostimulation. Mice on HFD showed reduced response to mechanostimulation as compared to mice on chow diet. **P≤0.01
(TIF)

Figure S2 **Spread of tracers in dermal lymphatic vessels in K14-VEGF-C mice.** NIR imaging was performed after intradermal injection of 5 μL of 25 μM P20D680 in K14-VEGF-C mice. Due to hyperplasia of dermal lymphatic vessels in this mouse strain, the tracer spreads throughout this superficial network of vessels rather than draining predominantly into deeper collecting lymphatic vessels. Representative mouse 5 minutes after injection (A) and 10 minutes after injection (B). Higher magnification (C) demonstrates that the tracer is contained within dermal lymphatic vessels.
(TIF)

Video S1 **NIR lymphatic imaging of afferent collecting lymphatic vessels draining into the popliteal LN in a FVB mouse after 17 weeks of chow diet.** Imaging was performed after injection of P20D680 (5 μL of 25 μM) into dorsal foot skin and uptake into collecting lymphatic vessels was observed. Video is 10x normal speed and was acquired with Cy5 filter set at 1 frame/s.
(MOV)

Video S2 **NIR lymphatic imaging of afferent collecting lymphatic vessels draining into the popliteal LN in a FVB mouse after 17 weeks of HFD.** Imaging was performed after injection of P20D680 (5 μL of 25 μM) into dorsal foot skin and uptake into collecting lymphatic vessels was observed. Video is 10x normal speed and was acquired with Cy5 filter set at 1 frame/s.
(MOV)

Video S3 **NIR imaging of lymphatic vessels in the lower limb in a K14-VEGF-C mouse after 17 weeks of chow diet.** Imaging was initiated 30 s after injection of P20D680 (5 μL of 25 μM) into dorsal foot skin. Spreading of the lymphatic tracer within dermal lymphatic vessels was seen. Video is 10x normal speed and was acquired with Cy5 filter set at 1 frame/s.
(MOV)

Video S4 **NIR imaging of lymphatic vessels draining in the lower limb in a K14-VEGF-C mouse after 17 weeks of HFD.** Imaging was initiated 30s after injection of P20D680 (5 μL of 25 μM) into dorsal foot skin. No spreading of the lymphatic tracer within dermal lymphatic vessels was seen. Video is 10x normal speed and was acquired with Cy5 filter set at 1 frame/s.
(MOV)
