# Supplementary figures and images for: Chronic High-Fat Diet Impairs Collecting Lymphatic Vessel Function in Mice
Source: PLoS One. 2014 Apr 8;9(4):e94713. doi: 10.1371/journal.pone.0094713 (PMC3979858; doi:10.1371/journal.pone.0094713)

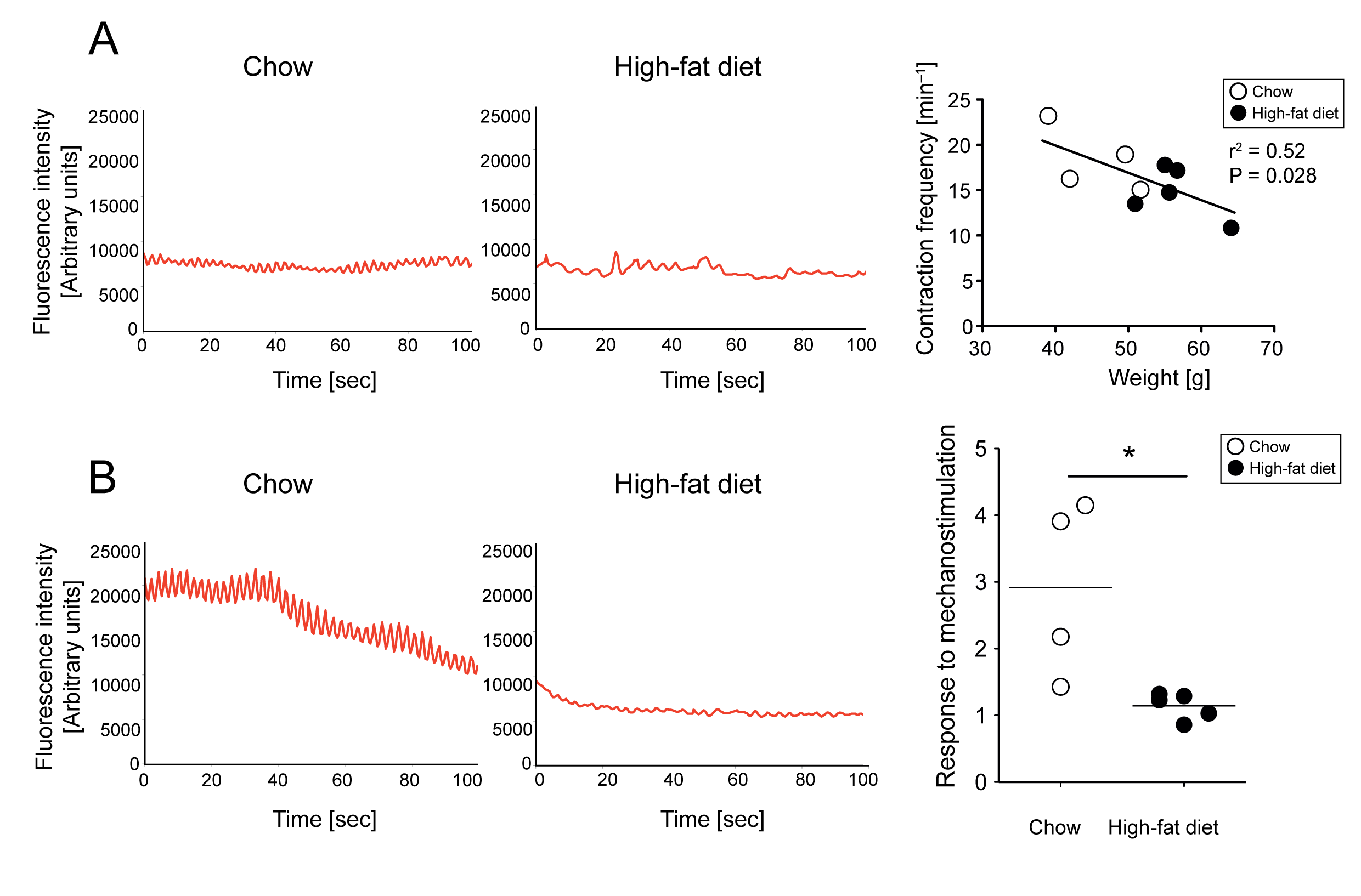

Supplement: Figure S1 — Impaired collecting lymphatic vessels function after 17 weeks of HFD in C57BL/6J:ICR mice. NIR imaging was performed after intradermal injection of 5 μL of 25 μM P40D680, a tracer specific for uptake into lymphatic vessels. (A) Normal and irregular contraction patterns in chow (n = 5) and HFD (n = 5) mice, respectively. Linear regression analysis shows a significant negative correlation between weight and contraction frequencies. (B) Normal and impaired response to mechanostimulation. Videos were initiated 15 s after mechanostimulation. Mice on HFD showed reduced response to mechanostimulation as compared to mice on chow diet. **P≤0.01 (TIF) [file pone.0094713.s001.tif]

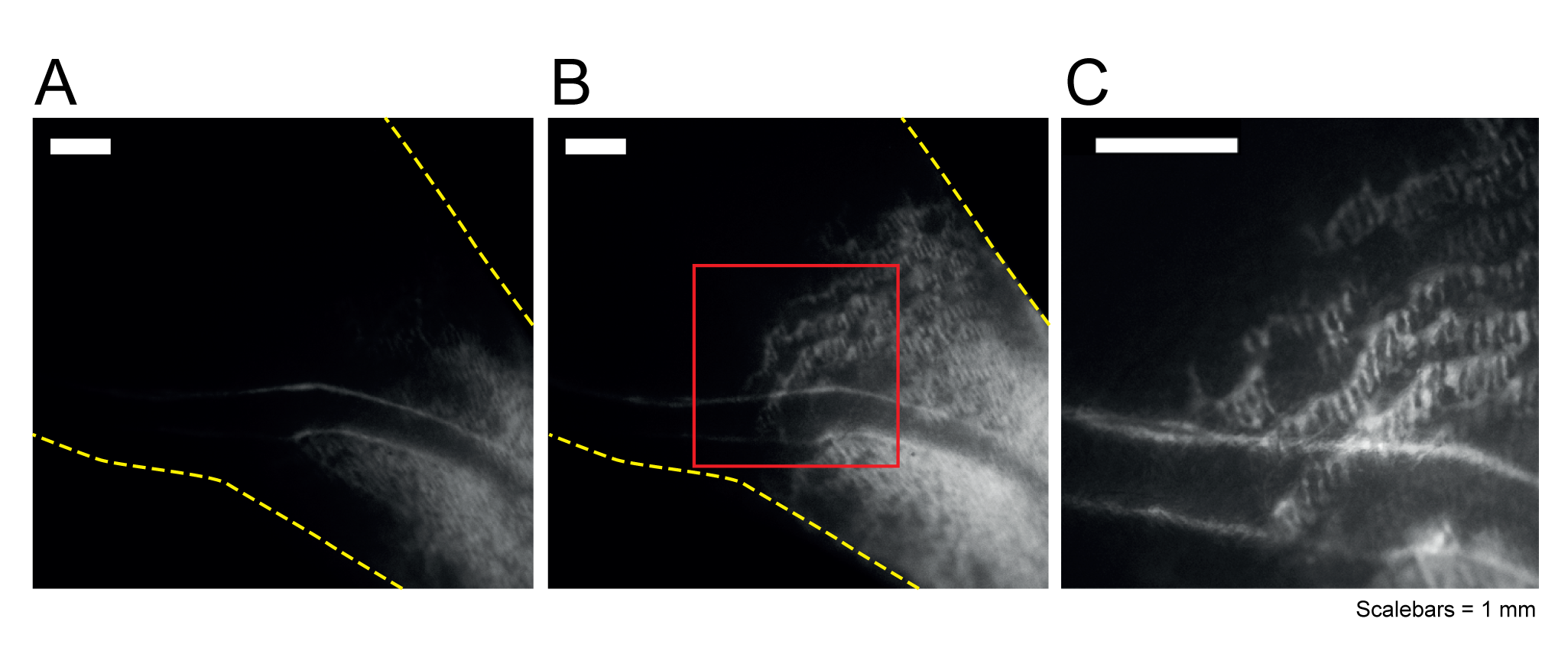

Supplement: Figure S2 — Spread of tracers in dermal lymphatic vessels in K14-VEGF-C mice. NIR imaging was performed after intradermal injection of 5 μL of 25 μM P20D680 in K14-VEGF-C mice. Due to hyperplasia of dermal lymphatic vessels in this mouse strain, the tracer spreads throughout this superficial network of vessels rather than draining predominantly into deeper collecting lymphatic vessels. Representative mouse 5 minutes after injection (A) and 10 minutes after injection (B). Higher magnification (C) demonstrates that the tracer is contained within dermal lymphatic vessels. (TIF) [file pone.0094713.s002.tif]
